# Adolescents with HIV and transition to adult care in the Caribbean, Central America and South America, Eastern Europe and Asia and Pacific regions

**DOI:** 10.7448/IAS.20.4.21475

**Published:** 2017-05-16

**Authors:** Heather Bailey, Maria Letícia Santos Cruz, Wipaporn Natalie Songtaweesin, Thanyawee Puthanakit

**Affiliations:** ^a^ Population, Policy and Practice Programme, UCL Great Ormond Street Institute of Child Health, University College London, London, UK; ^b^ Infectious Diseases Department, Hospital Federal dos Servidores do Estado, Rio de Janeiro, Brazil; ^c^ Department of Pediatrics, Faculty of Medicine, Chulalongkorn University, Bangkok, Thailand; ^d^ Research Unit in Pediatric Infectious Diseases and Vaccine, Chulalongkorn University, Bangkok, Thailand

**Keywords:** HIV, transition, youth, adolescents, loss to follow-up, adherence, outcomes, paediatric

## Abstract

**Introduction**: The HIV epidemics in the Caribbean, Central America and South America (CCASA), Eastern Europe (EE) and Asia and Pacific (AP) regions are diverse epidemics affecting different key populations in predominantly middle-income countries. This narrative review describes the populations of HIV-positive youth approaching adolescence and adulthood in CCASA, EE and AP, what is known of their outcomes in paediatric and adult care to date, ongoing research efforts and future research priorities.

**Methods**: We searched PubMed and abstracts from recent conferences and workshops using keywords including HIV, transition and adolescents, to identify published data on transition outcomes in CCASA, EE and AP. We also searched within our regional clinical/research networks for work conducted in this area and presented at local or national meetings. To give insight into future research priorities, we describe published data on characteristics and health status of young people as they approach age of transition, as a key determinant of health in early adulthood, and information available on current transition processes.

**Results and discussion**: The perinatally HIV-infected populations in these three regions face a range of challenges including parental death and loss of family support; HIV-related stigma and socio-economic disparities; exposure to maternal injecting drug use; and late disclosure of HIV status. Behaviourally HIV-infected youth often belong to marginalized sub-groups, with particular challenges accessing services and care. Differences between and within countries in characteristics of HIV-positive youth and models of care need to be considered in comparisons of outcomes in young adulthood. The very little data published to date on transition outcomes across these three regions highlight some emerging issues around adherence, virological failure and loss to follow-up, alongside examples of programmes which have successfully supported adolescents to remain engaged with services and virologically suppressed.

**Conclusions**: Limited data available indicate uneven outcomes in paediatric services and some shared challenges for adolescent transition including retention in care and adherence. The impact of issues specific to low prevalence, concentrated epidemic settings are poorly understood to date. Outcome data are urgently needed to guide management strategies and advocate for service provision in these regions.

## Introduction

The HIV epidemics in the Caribbean, Central America and South America (CCASA), Eastern Europe (EE) and Asia and Pacific (AP) regions are diverse, concentrated epidemics affecting different key populations, in predominantly middle-income countries. Adolescents (10–19 years) living with HIV numbered 74,000 in CCASA and 220,000 in AP in 2015, with 210,000 and 600,000, respectively, aged 15–24 years according to UNAIDS estimates [1]; the epidemic in EE and Central Asia is smaller (estimated 80,000 15–24 year olds), but the only one globally where overall incidence continues to rise substantially. Successes in prevention of mother-to-child transmission (PMTCT) have been uneven across countries in these regions [2–7] and alongside a perinatally HIV-infected (PHIV) population aging into adolescence and young adulthood [8–10], there is ongoing vulnerability of young people in marginalized key populations to behavioural HIV acquisition. Almost a third of new infections in CCASA in 2014 were in men who have sex with men (MSM), while in EE and Central Asia, over half of new infections are currently among people who inject drugs (PWID) [6]; young people within key population groups are particularly vulnerable to HIV acquisition and barriers to care [11,12].

Paediatric HIV research capacity and infrastructure is very limited in some of the most affected countries across CCASA, EE and AP. Paediatric HIV in these regions is at risk of being perceived as low priority compared with epidemics in key adult populations and much larger numbers of PHIV children living in sub-Saharan Africa, and this is reflected in a paucity of paediatric HIV research literature specific to these settings. For example, a recent systematic review of 2008–2013 data from low- and middle-income countries on paediatric retention in HIV care reported results from several large cohorts in Southern and Eastern Asia but only one in CCASA and none in Europe or Central Asia [13]. In countries with more mature epidemics, adolescence and the transition to adult HIV services has been associated with issues around retention and adherence, with outcomes of worsening immune status [14] and increased risk of virological failure [15,16]. PHIV and behaviourally HIV-infected (BHIV) young people in CCASA, EE and AP regions may face challenges specific to low prevalence, concentrated epidemic settings including issues around organization and level of healthcare resource and access, opportunities for peer support, and punitive treatment of most-at-risk populations.

This narrative review describes the populations of HIV-positive youth approaching adolescence and adulthood in CCASA, EE and AP, what is known of their outcomes in paediatric and adult care to date and ongoing research efforts and future research priorities.

## Methods

We searched PubMed and abstracts from recent conferences and workshops for published information on adolescent transition in CCASA, EE and AP using key words including HIV, transition and adolescents, to identify published data on transition outcomes. We also searched within our regional clinical/research networks for work conducted in this area and presented at local or national meetings. We focussed on studies exploring outcomes following transition to adult care (including clinical or immunological /virological outcomes, ARV resistance, loss to follow-up, death). Due to the paucity of data on post-transition outcomes, we also describe published data on characteristics and health status of young people as they approach age of transition, as a key determinant of health in early adulthood, and information available on current transition processes, to give insight into future research priorities.

## Results and discussion

### Characteristics of HIV-positive young people

PMTCT and paediatric treatment policies, both current and historic, require consideration in understanding the characteristics of the populations of PHIV young people who, alongside BHIV young people, require youth-orientated services in these regions.

#### Caribbean, Central America and South America

In CCASA, PMTCT programmes have been in place throughout the region since the late 1990s and around 74% of pregnant women have access to HIV testing and counselling; MTCT rates among diagnosed women in most affected countries are now around 4–5% [4]. The largest number of adolescents living with HIV are in Brazil and Mexico (28,000 and 6,600, respectively [1]). Many PHIV adolescents have been followed and treated since the first months of life. Nevertheless, late diagnosis of children still occurs; an observational study conducted at 15 paediatric sites in Brazil, Argentina, and Mexico analyzed data of 120 adolescents who acquired HIV from diverse sources, and found that 28% of the PHIV group were diagnosed at ≥10 years [17]. In this region, because of substantial HIV-related stigma, parents frequently avoid bringing older children to get tested or delay disclosure to young people of their HIV status [18].

CCASA has a large population of young people aged 15–24 years strongly affected by socio-economic inequalities, with more than 30% living in poverty [19]. Early sexual debut and adolescent pregnancy is common in the region (10% of women 15–19 years are mothers [19]), and data from Haiti have shown particular vulnerability of young women to sexual HIV acquisition [20]. Although the number of new infections among adolescents has declined in recent years and the estimated prevalence of HIV is below 1% overall in CCASA, prevalence is much higher among some key urban populations including young MSM (e.g. 13% among adolescent MSM in Paraguay [21,22]).

#### Eastern Europe (EE)

Eastern Europe’s HIV epidemic began in Ukraine in the mid-1990s, driven by injecting drug use (IDU) although with increasing infections over time in bridging populations (such as sexual partners of PWID) [23]. Currently, 8 in 10 new infections in EE and Central Asia are in Russia [6], which together with Ukraine accounts for around 90% of the people living with HIV (PLWH) in the region [24]. In Russia and Ukraine, antenatal prevalence is around 0.75% and 0.33%, respectively [25,26] and MTCT rates among diagnosed women are currently around 3–4% [23,27] having declined from 10% in 2003 in Ukraine [28] and around 5–8% in 2004–2005 in St Petersburg [29]. The region therefore has a comparatively young PHIV population; according to official figures, of 6454 PHIV children in HIV care in Russia in 2014, only 6% were aged over 14 years [27] (vs. 65% in the UK the same year [30]) while in the Ukraine Paediatric HIV Cohort study which covers around a third of PHIV children nationwide, median age at the beginning of 2016 was 10 years [IQR 6.9–13.0] [10]. In 2015, 97 young people transferred from paediatric to adult HIV care nationally in Ukraine at age 18 years [31] while there were 2764 children aged ≤14 years in HIV care [23]. PHIV children growing up in EE have high prevalence of exposure to maternal drug use (26% in the Ukraine Paediatric HIV Cohort [32]) due to poor PMTCT access among women who inject drugs [29,33] with potential for other related impacts on health – for example, hepatitis C co-infection, exposure to tuberculosis, and long-term sequelae of preterm delivery. Maternal IDU and perinatal HIV transmission have both been linked to infant abandonment [34], and high and increasing number of AIDS deaths in the adult population from 38,000 to 47,000 annually between 2010 and 2015 [35]) contribute to the number of PHIV children growing up without family support in EE. For those in foster or state institutional care [36,37], transition to adulthood may bring its own challenges, with abrupt shift in available support and responsibilities.

In Romania, a major iatrogenic HIV epidemic occurred in 1987 to the early 1990s among children who received unsafe injections and/or blood [38]; an estimated 10,000 children were infected and many survivors have been followed into adulthood [39,40]. Among new cases in young people in Ukraine and Russia, IDU continues to be an important mode of acquisition with injecting typically initiated in the late teens or early 20s [41,42] and limited coverage of harm reduction services [43]. Among street-involved youth, HIV prevalence of up to 28% has been recorded in the 15–24-year age group in Ukraine [37,44,45] with two-thirds in one study engaged in bridging behaviours with non-street youth [46]. There is also an epidemic among MSM which is largely absent from official figures [47,48] due to intense stigma and the hostile social/legal environment experienced by this group [49]. National data from Ukraine indicate a decline in the number of 15–24 year olds newly registering for HIV care from 34.5 to 18.9 per 100,000 between 2010 and 2015. However, less than half of PLWH are estimated to be diagnosed and late diagnoses with advanced disease are common [23].

#### Asia and Pacific (AP)

In AP, HIV PMTCT programmes were rolled out from the late 1990s; however, the regional coverage of ART for PMTCT in 2015 was only 41% [50]. Thailand is the only country in Asia to meet the goal of elimination of MTCT, with rates <2% in 2016, and a decline in antenatal prevalence from 2.3% in 1994 to 0.6% in 2015 [5]. Overall, access to ART among HIV-infected children and adults in the AP region began in the early 2000s [51] but with variable funding in each country from National budgets, the Global Fund to Fight AIDS, Tuberculosis and Malaria or the U.S. President’s Emergency Plan for AIDS Relief (PEPFAR). According to UNICEF, in 2015 there were more than 400,000 children orphaned to AIDS living in Indonesia, Thailand, Myanmar and Vietnam [50]. The TREAT Asia Pediatric HIV Observational Database (TApHOD) is a regional database including more than 4000 HIV-infected children and adolescents in Thailand, Cambodia, Malaysia, India, Indonesia and Vietnam. A report from the TApHOD database in 2011, at a time when one-third of children in the cohort were older than 12 years, showed that more than two-thirds of adolescents had lost at least one parent and the majority were cared for by grandparents or relatives [52]. Countries with the largest number of adolescents living with HIV in the region are India, Indonesia, Thailand, Myanmar, and Vietnam.

One of the challenges faced by PHIV adolescents transitioning to adult services in AP is that of not being fully disclosed of their diagnosis. The World Health Organization guideline recommends informing children of their HIV status between the ages of 6–12 years. However, many parents and care providers choose the easier option of letting adolescents ‘discover by themselves’ about their illness. A study among 260 children/adolescents in a paediatric HIV centre in Thailand showed that the median age of disclosure was 14.8 years [53]. PHIV adolescents usually have their medical care arranged by caregivers, and receive a one-stop service for HIV care. Therefore, they need preparation to understand their own health, be aware of their HIV status and be able to self-care and seek medical care in the adult healthcare system.

### Healthcare services and transition process

Examples of transition models are given in Box 1.

**Box 1.** Examples of different transition models

Jamaica
Children are routinely transitioned into adult care within the public sector at the age of 12 years, although at two paediatric sites transitioning happens as late as 24 years. Within the Jamaica Paediatric Perinatal and Adolescent HIV/AIDS Programme (JaPPAAIDS) transition is supported through involvement of a psychologist and intensive follow-up; there are plans to rollout this model to other sites in Jamaica [54].
Ukraine
HIV care for children and adults is provided at regional HIV centres; transfer to adult care involves a change of doctor within the same centre and usually occurs at 18 years, with some flexibility depending on the policy of the centre. Transfer is normally preceded by a period of shared care between the paediatrician and adult doctor [31].
Thailand
At Chiangrai Prachanukroh Hospital in Chiang Rai province, a group transition programme started in 2008. Young people who are ready to transfer are selected by paediatric providers and case conferences held with adult providers, the hospital’s home care team and clinical psychology consultants. The young people attend a camp for 1–2 days to prepare them for transition and then attend adult clinic as a group, accompanied by a paediatric provider for the first few appointments [55].

#### Caribbean, Central America and South America (CCASA)

In CCASA, many paediatric HIV clinics have established dedicated clinical settings for the adolescent population and most provide care for adolescents regardless of mode of acquisition with many BHIV adolescents referred from sexually transmitted disease and pregnancy clinics. Programmatic data from Haiti demonstrate the positive impact of youth-friendly adolescent HIV services on the proportion of young people initiating ART [56]. A proportion of youth living with HIV in CCASA are already parents [57], most of whom are still being followed at adolescent clinics, where they have, many times, built strong bonds.

Data from CCASA indicate that PHIV or BHIV adolescents receiving care in adolescent clinics, as well as their healthcare professionals, may perceive the prospect of transitioning to adult clinics as a threatening event which entails a rupture in relationships [58,59] or be intimidated by the atmosphere of an adult clinic [54]. In an example of a model aiming to support the transition process at an Adolescent Clinic in São Paulo, Brazil, readiness of young people to transition is evaluated (e.g. clinically and emotionally stable, adequate social support, and knowledge about HIV and ART) along with preparation in paediatric services followed by more intensive follow-up in adult services in the year following transition [60]; qualitative findings from young people post-transition showed that some reported insecurity in relationships with adult care providers and in expectations for the future [60]. Unfortunately, in CCASA, most specialized health services are not well connected with community-based and educational resources that could help transform life perspectives and empower youth for adult life. Strategies such as case management are not routinely available in services of the region.

#### Eastern Europe (EE)

In Russia, Ukraine and some other countries of the former Soviet Union, HIV care for children and adults is delivered through a network of regional HIV centres. Linkages to other services (e.g. for TB treatment) are often weak [61]. However, progress has been made to decentralize ART provision in Ukraine in recent years [62]. Typically, HIV-positive children in Ukraine are cared for by a paediatrician until 18 years, when they transition to adult services within the same centre [31]. In Russia, some centres follow a similar system while others follow a family-based model whereby young people continue to be seen by the same infectious diseases doctor into adulthood (personal communication, Anna Turkova 2016). This model is also followed by the “Dr Victor Babes” Hospital for Infectious and Tropical Diseases Bucharest, one of the HIV referral centres in Romania (personal communication, Luminita Ene, 2016). Where paediatric and adult HIV care are provided by different specialists, the setting in which a young person begins their care is determined by age at diagnosis; issues around transition of care therefore apply to BHIV young people diagnosed aged <18 years as well as PHIV. However, delays in linkage to HIV care following diagnosis are particularly long for PWID (mean 3 years) and young people [63] and the proportion of BHIV entering paediatric HIV services is therefore likely to be quite small. ART access for children has been prioritized in EE and currently ART coverage is >90% in Ukraine and 75% in Russia [10,23,27] vs. around 50% and 34% among adults, respectively [23,64]. Access to some aspects of HIV care such as ARV resistance testing is poor or uneven [61] and young people and their families may face additional barriers including illegal charging of users fees for non-HIV-specific services [65–68] and poor access to sexual and mental health services [69,70].

#### Asia and Pacific (AP)

In AP, the process of transition depends on the clinical setting; large hospitals such as university hospitals or regional hospitals have separate paediatric and adult HIV clinics, therefore formal patient transfer between clinics is needed. In general, the cutoff age for transfer from paediatric to adult services is 15 years. In smaller service delivery centres such as provincial hospitals, healthcare workers must provide HIV care to all groups of patients, including pregnant women, children and adults, therefore, transition to adult care is implemented only through an adjustment of the mindset and the approach healthcare workers employ with patients once they reach adolescence.

The majority of healthcare providers surveyed within the TREAT Asia network in December 2012 felt the most appropriate age of transition to be between 18–20 years [71]. The main barriers for transition to adult care included perceived lower quality of care in adult services, less adolescent-friendly HIV care settings, and less expertise in adolescent psychosocial needs. Solutions suggested by survey respondents for smooth transition included disclosure of HIV status, preparation for transition 1–3 years in advance, preparation of up-to-date medical summaries, identifying adult HIV clinicians with an interest in the transitioning population and assignment of staff to oversee the transition process. Other supportive measures included engaging the multidisciplinary team at both paediatric and adult levels, maintaining relationships with transitioned adolescents, and encouraging the patient’s family to take responsibility within the transition process. An interview among 19 HIV-infected Thai youth regarding the transition process, 9 of whom had already transitioned, found that they were fearful of the sudden increase in responsibility when navigating through adult HIV services with less psychosocial and adherence support, and of disclosure of details of their infection to other adults [71]. The group brainstormed solutions to alleviate these fears, such as education about medications, how to manage medical appointments, and preparation on communication skills and building relationships with adult healthcare service providers.

Several initiatives in AP have aimed to empower adolescents living with HIV and prepare them to become independent young adults. A study conducted in Vietnam asking healthcare workers about concerns of adolescents living with HIV found an urgent need for education about sexual health and disclosure [72]. UNESCO in collaboration with the Ho Chi Minh Communist Youth Union and the Ministry of Education and Training produced “As We Grow Up” and “Happy living with ‘H’” booklets, which provide adolescents with knowledge on healthy sexuality and lifestyles, HIV knowledge, strategies for dealing with stigma and discrimination, as well as coping skills in an adolescent-friendly format. Between 2014 and 2015, several national training workshops for healthcare workers were held throughout Vietnam to introduce the concept of transition and to launch these booklets in an effort to increase support for adolescents living with HIV. A 2015 intervention programme in Thailand designed for adolescents called the “Happy Teen Program”, which focused on improving health knowledge, coping skills, sexual risk reduction and life goal setting, was piloted and implemented using individual counselling sessions and group work [9].

### Outcomes in paediatric and adolescent care

#### Caribbean, Central America and South America (CCASA)

Data from the Caribbean, Central and South America Network for HIV Epidemiology (CCASAnet) cohort on 903 adolescents living with HIV in Argentina, Brazil, Chile, Haiti, Honduras, Mexico and Peru [8], of whom 53.8% were female, indicated that at last study visit 90% were on combination ART (cART), median CD4 count was 598 cells/mm^3^ (IQR 377–860) and 70% were considered virologically suppressed (<400 copies/mL) with median WHO height-for-age z-score of −1.59. Mortality between age 10 and 15 years was 4.4% (95%CI 3.1–6.1). A multicentre Brazilian survey among 260 children and adolescents receiving routine care at referral centres found 57% of children and 49% of adolescents with viral suppression (<50 copies/ml) [73]. This same study observed that at entry to paediatric HIV care, 44.6% of children had already progressed to either an AIDS defining illness or CD4 percentage <15%. In another study of 387 children aged 1–11 years from Brazil, Peru and Mexico, 57.6% had a viral load <400 copies/ml [74]. There are no available data on neuropsychological aspects or cognition among adolescents living with HIV in CCASA; however, a Brazilian study found a high frequency of school failure and drop-out (51% and 28.6% respectively) among adolescents living with HIV in the northeast region of the country [75]. Non-adherence to treatment is frequent, sometimes indicating psycho-social situations (e.g. lack of self-efficacy) which need attention [76], while family social isolation, stigma (and resulting issues around disclosure [77]), anxiety and depression, and substance misuse are examples of common challenges that affect treatment outcomes of children and adolescents. Some of these situations can be identified and addressed during follow-up of adolescents and their caregivers [73,78,79].

#### Eastern Europe (EE)

In EE, data on outcomes in paediatric care are available within the European Pregnancy and Paediatric HIV Cohort Collaboration (EPPICC) from the Ukraine Paediatric HIV Cohort set up in 2011, and for Russian sites in St Petersburg and Irkutsk which have participated in EPPICC since 2012. Analyses within EPPICC have indicated that children in these two countries have poorer response to cART than children in Central /Western Europe, with a significantly lower probability of reaching virological suppression of <400 copies/ml by 12 months after cART initiation (aHR 0.73, 95% CI 0.64–0.84, after adjusting for other factors including age and calendar year at cART initiation) [80]. This finding may be partly due to targeted viral load testing of children with poor CD4 response or clinical signs of treatment failure, in settings with limited viral load testing capacity; results from the Ukraine cohort indicate good rates of virological suppression of 88% at last follow-up among both older (≥14 years) and younger children on a stable ART regimen for the preceding 6 months [10], similar to the 92% (95% CI 91–94) virological suppression rate seen by 12 months among 997 children in the UK/Ireland cohort [81]. EPPICC analyses have also indicated that children in Ukraine and Russia are slower to switch to second-line therapy than those in Western Europe [82], possibly due in part to poorer availability of second-line drugs. Studies in Romania indicate low rates of school completion and high prevalence of neurocognitive dysfunction among HIV-positive adolescents [39,40], with greater risk of HIV disease progression and/or death among those whose HIV status was not disclosed [83] or in families with lower socio-economic status [84].

#### Asia and Pacific (AP)

In AP, data from the TREAT Asia network indicate that in paediatric services, more than 80% of treated adolescents had a good virological outcome (<400 copies/ml) [52]. Aurpibul in 2015 reported on a cohort of 107 Thai children enrolled in an ART programme since 2002; at 10 years follow-up (median current age, 17.8 years), 8% had died, 8% had been referred to decentralized ART services, 84% remained in follow-up, 77% had viral suppression and 71% had CD4 counts of >500 cells [85]. Data on neurocognitive function among older children in Thailand and Cambodia showed that HIV-infected children had lower cognitive function compare to HIV-negative children (mean full scale IQ 75 versus 90) despite receiving cART, which may in turn affect their ability to self-care and transition to adult clinic [86].

### Transition outcomes: current data and ongoing research

Published data on outcomes of young people transitioning to adult HIV care in CCASA, EE and AP are very limited to date. This is due to the comparatively young PHIV population in some countries across these regions and the historical lack of paediatric studies and surveillance data with sufficient duration of follow-up in some of the most affected countries, which provide the basis for tracking young people into adult care. The main findings from five published studies on transition outcomes across these regions are given in Table 1. The studies highlight some common issues including poor rates of retention in care and suboptimal virological suppression due to ongoing poor adherence and/or exhaustion of treatment options.

**Table 1.** Transition outcomes of HIV-positive young people transferring to adult care in the Caribbean, Central America and South America, Eastern Europe and Asia and Pacific

Region	Country	Year of study	Study population	Main findings	Ref
CCASA	Argentina	2015	37 perinatally infected adolescents transitioning to adult care centre “Cosme Argerich” Hospital, Buenos Aires, Argentina in 2005–2011	Of the 37 who transitioned in this time period, 62% (n = 23) had ongoing virological failure Among 11 of the 23 adolescents included in a detailed study on virological failure, 10 had triple-class experience and five had triple-class resistance	[87]
CCASA	Brazil	2016	41 young people (39 perinatally infected) transferring to adult HIV care at an HIV outpatient clinic in Sao Paulo, median age 19 years at first adult clinic visit.	Median CD4 count at first adult clinic visit was 250 cells/mm^3^ (IQR 94–460) and 46% (n = 19) had a viral load <400 copies/ml Around 70% had adherence failure in the final two years of paediatric care (according to a composite measure including missed ART and/or clinic appointments) and a similar proportion in the first two years of adult care.	[88]
EE	Russia	2016	20 adolescents (18 years) who transferred to adult care in St Petersburg in 2013–2015, 19 behaviourally and 1 perinatally infected	By 2016, 3 remained in active follow-up; 4 had been referred to other centres; 13 were lost to follow-up	[89]
AP	Thailand	2015	67 young people transitioning from paediatric to adult care at Chiangrai Prachanukroh Hospital between 2008–2014 at >18 years of age	By March 2015, 73% (n = 49) remained in active follow-up; 13% (n = 9) had been lost to follow-up; 7% (n = 5) had been referred to other hospitals; 6% (n = 4) had died. Two of four deaths took place within 8 months of transitioning and two deaths after >2.5 years. All four deaths were in patients with non-suppressed viral load at time of transition to adult care. Of the 49 remaining in active follow up in 2015, 76% had a viral load <40 copies/ml.	[55]
AP	Malaysia	2016	21 adolescents who had transitioned to the adolescent transition clinic at Institut Pediatrik, Hospital Kuala Lumpur (a tertiary referral centre) in 2010–2015	Only one of the 21 patients had been lost to follow-up by 2016 Some patients required switching of their cART due to treatment failure, however there were few risky health behaviours and all patients were doing well and in employment	[90]

#### Caribbean, Central America and South America (CCASA)

A study from Argentina highlighted substantial treatment experience of adolescents at the point of transition to adult care; among 11 young people with virological failure, median treatment duration was 15 years (IQR 12–16) with median 3 ART regimens (IQR 3–9) [87]. In a study in Brazil, three-quarters of the 41 young people had received mono or dual therapy before beginning cART, with a median 4 previous ART regimens by the time of transfer to adult care [88]. Problems with adherence and retention in paediatric care persisted following transition to adult care (Table 1).

Within CCASAnet, planned studies of HIV-positive youth include two projects soon to begin on pregnancy outcomes in young PHIV and BHIV women, to include investigation of retention in care after delivery (personal communication, Catherine McGowan, 2017).

#### Eastern Europe (EE)

The only study from EE was among 19 BHIV and 1 PHIV 18 year old who transferred from paediatric to adult HIV care in St Petersburg, Russia in 2013–2015; very high rates of loss to follow-up (13/16 among those who remained in the region) were reported [89]. However, the barriers to retention in adult care experienced by sexually infected youth may differ from those faced by PHIV adolescents (who comprise the majority of those in paediatric care). Data from the adult population in EE highlight particular adherence challenges faced by young women, with over half of those aged <25 years in a Ukraine cohort reporting that they had missed a dose in pregnancy compared with only 23% aged 25–27 years [91].

Data collection on outcomes of young people in adult HIV services (including those transferring from paediatric care) is ongoing in Ukraine, within a research project building on the Ukraine Paediatric HIV Cohort and funded by the International AIDS Society’s Collaborative Initiative for Paediatric HIV Education and Research (CIPHER) programme. This project includes a computer-assisted self-interview of 13–24 year olds at two sites to support incorporation of their needs into transitional programs. More broadly within EPPICC, research currently in development aims to characterize the population at transition and the subsequent outcomes.

#### Asia and Pacific (AP)

A study from Thailand evaluated transition outcomes of 67 adolescents who prepared for transition through a two day camp where they were introduced to the adult care team in groups of 10–15, before transitioning to the adult HIV clinic in the same hospital (Table 1 [55]). Five years post transition, the majority remained in follow-up care with good virological outcomes. It was found that having adolescents visit adult services with the paediatric team facilitated positive peer interactions that provided resilience against poor drug compliance, alcohol use, difficult family relationships as well as other life issues. In Malaysia, a structured, multidisciplinary transition programme at the Institut Pediatrik Hospital Kuala Lumpur, a large tertiary centre, also demonstrated good outcomes with only one patient of 21 lost to follow-up in five years of follow-up [90].

The Study of Transitioning Asian Youth (STAY) is aiming to describe transition outcomes of PHIV adolescents in terms of retention in care, adherence to ART and virological suppression rate after transfer to adult care with annual visits and biannual online/mobile surveys [54]; Enrolment of 100 HIV-infected adolescents in Thailand, Malaysia, and Vietnam into this study started in 2016 (personal communication, Annette Sohn, 2017).

### Future research priorities

Across CCASA, EE and AP, there are key evidence gaps regarding development of evidence-based clinical practice guidelines tailored to the country context to support transitioning youth. Increasing numbers of young people are anticipated to transition to adult care in the coming years against a backdrop of increasing numbers of adults on treatment, as the WHO 2015 guidelines recommending ART for all PLWH are incorporated into national guidelines, with implications for burden on adult services. The period of 18–25 years of age, presently understood in developed countries as “emerging adulthood” [92], is likely to be experienced differently among HIV-positive youth in CCASA, EE and AP compared with high-income or high prevalence settings, but HIV services and/or research for children and young people are sometimes given low priority within national policy agendas [93]; data are needed to inform advocacy for this group.

As in other regions, robust monitoring and longitudinal research studies on PHIV and BHIV adolescents are urgently needed to expand the currently very sparse data on transition outcomes such as retention rates, virological suppression and patient quality of life, and to guide future management strategies. The diverse models of healthcare for HIV-positive young people and different initiatives to support them that we describe highlight the need to establish common definitions and outcome measures, to facilitate within and between country comparisons. Young adulthood is a time of geographical mobility, and structural barriers to HIV care imposed by insurance or programmatic constraints may also be barriers to research. For example, HIV care in Thailand is supported by the national health security office for the general population, and the social security office (SSO) for those in employment. As adolescents begin to enter the workforce, their healthcare is transferred to the SSO, which can only be received at specific hospitals, often forcing them into transition without a preparation phase. This may compromise their outcomes and research efforts to follow them post-transition. In Ukraine and Russia, follow-up of HIV-positive young people from paediatric to adult care is possible within regional HIV centres; however, conflict in the East of Ukraine had resulted in displacement of >1.7 million people, including 228,000 children [94], and consideration needs to be given to the research challenges of tracking this vulnerable population within national HIV care systems. National data disaggregated by adolescent and young adult age groups are unevenly available across these regions but are crucial for the planning of services.

Long duration of ART with exposure to sequential changes in regimens and accumulation of resistance mutations is a special challenge for professionals caring for PHIV young people [57,87,95–97]. Work in CCASA has demonstrated the importance of ensuring a comprehensive approach to adolescent HIV care in which factors affecting treatment outcomes can be identified and addressed before the transitioning process begins [73,78,79]. Peer support may improve outcomes [55,98]; however, young people across CCASA, EE and AP who are part of stigmatized or criminalized groups including MSM, sex workers, PWID or their sexual partners may face particular challenges in accessing this. Many adolescent clinics in CCASA are admitting MSM youth who subsequently identify as transgender females. Some paediatricians have personal and technical difficulties in following these patients and the transitioning process in these cases may occur prematurely. This is a very sensitive and challenging situation that deserves attention; transgender youth may face personal crisis during the period where transition is expected to occur and the non-observance of their specific needs may have direct impact on (future) treatment adherence and retention in care. Further work is needed to understand how the linkage of healthcare services to youth-centred community-based initiatives, particularly professionalizing resources, may facilitate social support and professional integration alongside the transitioning process and support post-transitioning outcomes.

## Conclusions

Limited data available from CCASA, EE and AP to date indicate uneven outcomes in paediatric services and some shared challenges during adolescence and transition, including retention in care and maintenance of virological suppression against a backdrop of adherence problems or exhaustion of treatment options. The impact of other context-specific challenges on young people in these regions are poorly understood to date. A range of transitional care models are currently being implemented and/or developed in countries across the regions, and there is an urgent need for setting-specific outcome data to inform development of resilient and sustainable systems which can support young PLWH during adolescence and young adulthood in years to come.
